# Blood–Brain Barrier Modulation to Improve Glioma Drug Delivery

**DOI:** 10.3390/pharmaceutics12111085

**Published:** 2020-11-12

**Authors:** Huilong Luo, Eric V. Shusta

**Affiliations:** 1 Department of Chemical and Biological Engineering, University of Wisconsin-Madison, Madison, WI 53706, USA; hluo67@wisc.edu; 2 Department of Neurological Surgery, University of Wisconsin-Madison, 600 Highland Avenue, Madison, WI 53792, USA

**Keywords:** blood–brain barrier, drug delivery, glioma, glioblastoma, targeting

## Abstract

The blood–brain barrier (BBB) is formed by brain microvascular endothelial cells that are sealed by tight junctions, making it a significant obstacle for most brain therapeutics. The poor BBB penetration of newly developed therapeutics has therefore played a major role in limiting their clinical success. A particularly challenging therapeutic target is glioma, which is the most frequently occurring malignant brain tumor. Thus, to enhance therapeutic uptake in tumors, researchers have been developing strategies to modulate BBB permeability. However, most conventional BBB opening strategies are difficult to apply in the clinical setting due to their broad, non-specific modulation of the BBB, which can result in damage to normal brain tissue. In this review, we have summarized strategies that could potentially be used to selectively and efficiently modulate the tumor BBB for more effective glioma treatment.

## 1. Introduction

**Figure 1:**
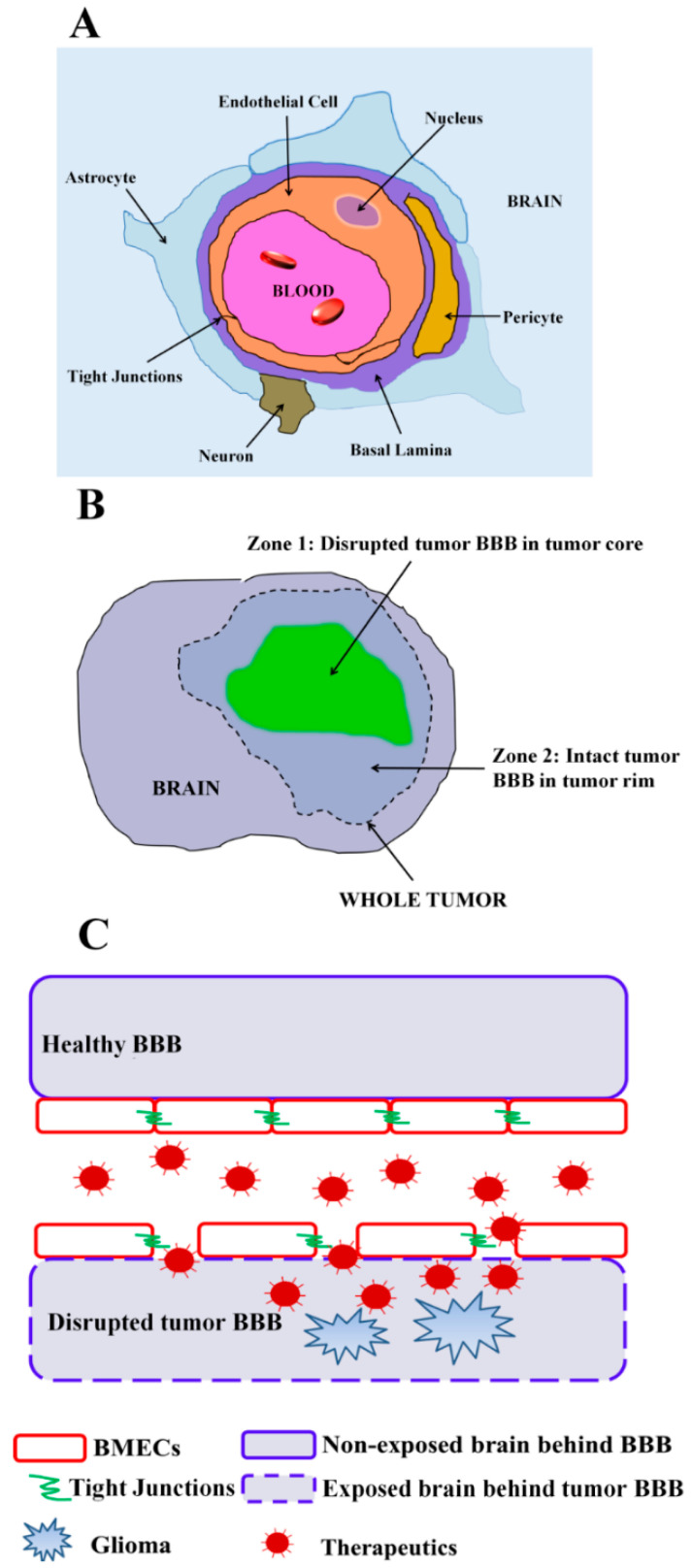
(**A**) Cross-sectional view of the neurovascular unit with the brain endothelial cells forming the blood–brain barrier (BMECs). Brain endothelial cells are connected by adherens and tight junctions, share a basement membrane with pericytes, make contacts with astrocyte endfeet and respond to neuronal cues. (**B**) Cartoon representation of the heterogeneity of tumor BBB permeability in glioma. Two zones can often be observed within the tumor volume—zone 1: disrupted tumor BBB in the tumor core imaged via MRI contrast leakage; zone 2: intact tumor BBB in the tumor rim area. (**C**) Cartoon depiction of drug delivery through normal and pathologically disrupted tumor BBB. Under normal conditions, the healthy and intact BBB separates the CNS from blood components, thereby preventing therapeutics from accessing the CNS. Under conditions in which glioma induces pathologic tumor BBB disruption, therapeutics can access the CNS via passive penetration or actively targeting the exposed components behind the disrupted tumor BBB.

Gliomas are one of the more frequent tumors occurring in the central nervous systems (CNS), accounting for over 32% of primary CNS cancers and over 77% of primary malignant brain cancers [1]. Gliomas include glioblastoma (GBM), a highly-aggressive brain tumor characterized by its rapid proliferation and angiogenesis, leading to a high mortality and making it the leading cause of cancer-related death in young adults aged 20–39 years and the second leading cause of death in children with cancer [2]. The prognosis of GBM patients is quite poor, with a median survival of approximately 14.6 months [3], and 5 year and 10 year survival rates of 5.0% and 2.6%, respectively [4]. Despite these challenges, new therapeutic regimens have been few. Although many new highly potent cytotoxic agents have been developed, systemic administration of these drugs has not increased the median survival of glioma patients in large part due to their poor BBB penetration [3]. The BBB is a highly specialized vascular interface that protects the neuronal environment from most blood-borne materials, and oftentimes the BBB excludes therapeutics. The BBB is formed by brain microvascular endothelial cells (BMECs) that work in concert with other cells of the neurovascular unit such as pericytes, astrocytes and neurons to regulate the movement of molecules and cells between blood and brain (Figure 1A). BMECs are non-fenestrated endothelial cells connected by junctional complexes that include adherens junction proteins (AJ, such as VE-cadherin) and tight junction (TJ) proteins, such as claudins, occludin and zonula occludens protein 1 (ZO-1). These properties, along with low levels of vesicular trafficking, help form a physical barrier to diffusion of material from the blood into the brain. Brain accumulation of hydrophilic small molecules and large-molecule biologics, such as siRNA/mRNA, monoclonal antibodies and antibody–drug conjugates are significantly blocked by this physical barrier [5,6]. For lipophilic small molecules that can diffuse across the plasma membranes of BMECs, efflux transporters expressed in BMECs often pump the drugs back into the bloodstream, acting as an active barrier [7]. These passive and active barrier properties presented by the BBB make it difficult to achieve therapeutic drug concentrations when treating glioma. Thus, it is of importance to enhance drug delivery across the BBB in order to improve glioma therapy and prognosis.

Despite the substantial barrier imparted by the BBB, its integrity can be disrupted by several pathologic conditions, including glioma. In the clinic, BBB disruption in glioma has been observed by accumulation of gadolinium-based magnetic resonance imaging (MRI) contrast agents within tumor regions [8]. A significant leakage of Evans blue and gadolinium at the glioma site also indicates BBB disruption [9]. This leaky glioma BBB caused many to suggest that the BBB is no longer limiting drug delivery and hence efficacy of therapies in treating GBM [5]. However, any drug delivery enhancement that results from BBB leakage is not enough to cure GBM. In fact, even when drugs help treat the BBB-disrupted tumor bulk or when neurosurgeons perform a gross total resection of all BBB disrupted regions of the tumor core indicated by contrast-enhancing agent, the tumor will recur within months in nearly all of these GBM patients [10]. While there is BBB disruption in GBM, numerous studies have shown that this disruption is heterogeneous in GBM patients [11,12,13,14]. As a result of the heterogeneous disruption and the highly-invasive nature of GBM, tumors can be found in areas with an intact BBB in the tumor rim (Figure 1B), where this intact BBB can still limit the distribution of therapeutic drugs [15]. As such, in this review, we will refer to the heterogeneously permeable vasculature of the tumor including both the tumor core and rim as the “tumor BBB”. Because of the tumor lying in areas behind the comparatively healthy tumor BBB in the tumor rim and invasive margins, many novel glioma therapeutics have failed in the clinic, in part because of the inability to reach effective drug concentrations [7]. Thus, despite tumor BBB disruption in the tumor core, it is still essential to establish strategies that can mediate delivery of therapeutic agents to the tumor rim and invasive margins when treating GBM.

A variety of strategies have been suggested to overcome the tumor BBB, thus improving drug delivery in treating glioma [16]. These include approaches to increase drug permeability through chemical modification, inhibition of efflux transporters, transcytosis via targeting of endogenous BBB transporters/receptors, and osmotic BBB disruption, among others and these have been reviewed elsewhere [16,17,18]. Such strategies can often lead to broad drug distribution in the brain, which can result in damage to the non-diseased tissue. For example, while osmotic disruption has met partial clinical success for the treatment of GBM, non-selective opening of the BBB induced by mannitol can also result in various complications including epilepsy and brain edema [19]. Thus, selective and efficient opening of the tumor BBB at the site of glioma would be beneficial for the targeting of cytotoxic therapies and minimization of side effects [10]. In this review, we will discuss drug delivery strategies that hinge on selective targeting of pathological tumor BBB disruption or selective biochemical or physical modulation of tumor BBB permeability to allow for enhanced, localized drug uptake to glioma.

## 2. Leveraging Pathological BBB Disruption in Glioma

As described above, tumor BBB disruption in glioma is heterogeneous and is quite dependent on the pathological development or stage of glioma [20]. In addition to gadolinium-contrast MRI, the permeability of ^14^C-sucrose in rat GBM area was 25-fold higher than that in the normal brain tissue [21]. Furthermore, intracarotid injection of anti-tumor drug, cisplatin, demonstrated a 10-fold higher distribution in GBM area compared with the control normal brain tissue [21]. Increased tumor BBB disruption tends to correlate with higher-grade or more malignant glioma in human patients [22], and a systematic study in mice using real-time MRI indicated that the tumor BBB remains intact in earlier glioma stages but becomes significantly disrupted in the tumor core in late stages of glioma progression [23]. In part, this is a result of glioma malignancy grade being positively correlated with angiogenesis, leading to more immature and higher permeability vessels within the tumor [24,25]. Thus, tumor BBB leakiness in the pathological progression of glioma has been used as a surrogate target for selective drug delivery into the tumor site during therapy [26,27]. To date, two main treatment strategies have been explored to exploit tumor BBB leakiness in the tumor core, one that relies on passive drug accumulation at sites of tumor BBB disruption and another that leverages specific targeting of glioma regions with disrupted tumor BBB.

### 2.1. Passive Drug Accumulation at Sites of Tumor BBB Disruption

First, the ability for normally BBB-impermeant drugs to accumulate at sites of tumor BBB disruption is suggested by the selective accumulation of gadolinium-based MRI contrast agents (normally 500–1000 Dalton) in the tumor bulk in areas of disrupted tumor BBB [8]. It is therefore also possible to deliver drugs directly to the leaky tumor regions with similar molecular weight or size. Recently, Mittapalli and colleagues employed quantitative fluorescent microscopy in a rat GBM model, in which simultaneous administration of multi-sized tracers (up to 625 kDa) were used to determine vascular permeability [28]. Three molecules ranging from 100 to 70 kDa penetrated into the GBM bulk at rates similar to their diffusion in water, suggesting that these solutes freely diffuse from the blood to the GBM site across disrupted vascular pores without steric restriction, and calculated pore diameters were >140 nm [28]. In another study, a panel of differentially-sized nanoparticles was used to measure the tumor BBB permeability threshold in an RG-2 glioma model and it was found that 330 kDa and smaller nanoparticles were able to pass the disrupted tumor BBB and enter the tumor [29]. Despite the core tumor BBB leakiness, small-molecule drugs typically fail to accumulate to therapeutic concentrations [10]. The challenge is even more substantial for those drugs that are substrates for efflux transporters present both at the tumor BBB and in tumor cells. For instance, approved anti-tumor drugs for treating GBM such as temozolomide, topotecan, etoposode, irinotecan and vincristine are substrates of various efflux transporters expressed on the membrane of GBM cells and BMECs [10,30,31,32,33]. To help increase the concentration of drugs at sites of disrupted tumor BBB and overcome drug efflux transporters, nanoparticles loaded with anti-tumor drugs have been deployed [33,34]. Importantly, the pore size of the tumor BBB openings in the tumor core can be as large as 500 nm [35], which can allow nanoparticles to traverse the disrupted BBB and accumulate in tissue. The most-studied nanoparticulate systems are liposomal in nature. Liposomal encapsulation of many anti-cancer drugs such as doxorubicin have proven efficacious in animal models of GBM [36,37], and multiple clinical trials [38,39,40,41,42] have tested liposome-loaded doxorubicin for treatment of malignant glioma. Unfortunately, despite the promise of such formulations, reliance on passive diffusion and accumulation of drug-loaded liposomes has not yet produced durable therapeutic outcomes in the clinic.

### 2.2. Targeted Drug Delivery at Sites of Tumor BBB Disruption

A potential strategy to further the accumulation of drugs in the disrupted region is to enhance the retention of drugs. The normally non-exposed brain tumor tissue behind an intact BBB would be exposed after pathological disruption of the BBB in the tumor core. This exposure would render brain extracellular matrix (ECM) and cellular components in the tumor microenvironment accessible to circulating therapeutics, offering opportunities for selective targeting to the tumor BBB-disrupted glioma region (Figure 1C). For instance, our laboratory has identified targeting molecules derived from variable lymphocyte receptors that target both brain ECM and brain tumor ECM. P1C10, the lead candidate, specifically accumulated in regions of orthotopic GBM tumors with disrupted tumor BBB, with minimal uptake in normal brain or peripheral organs [43]. Importantly, P1C10-targeted doxorubicin-loaded liposomes selectively accumulated in tumor BBB-disrupted regions, significantly improving survival over mock-targeted controls, indicating the value of targeting and retention in the disrupted regions [43]. In addition to brain ECM, many other components are also exposed after tumor BBB disruption, including but not limited to components from astrocytes, pericytes, microglia and glioma cells themselves. For example, receptors that are highly expressed on the surface of glioma cells can allow for targeted delivery of agents into tumor BBB-disrupted regions, including interleukin-4 receptor (IL-4R) [44], interleukin-13 receptor (IL-13R) [45,46,47] and neurokinin-1 (NK-1) [48]. For example, CRKRLDRNC peptide (AP1 peptide), selected via phage-display technology, was reported to selectively bind to IL-4R [28]. This tumor-homing peptide, AP1, can target doxorubicin-loaded nanoparticles to accumulate in glioma to a larger extent than untargeted nanoparticles, and achieve better therapeutic outcomes in glioma-bearing mice [44]. Similarly, a peptide derived from IL-13 protein was established to be able to specially bind with IL-13Rα2, which was highly expressed on GBM cells but not on normal brain tissues [45,46,47]. This IL-13 peptide was used to target docetaxel-loaded nanoparticles, increasing uptake in orthotropic GBM tumors [49]. Some other successful attempts to utilize the IL-13-targeting peptide to enhance delivery of GBM therapy have also been reported [47,50,51,52,53,54]. Neurokinin-1 (NK-1) receptors were also found to be selectively overexpressed in several malignant tumors including GBM [48]. Substance P peptide (RPCPQQFFGLM), one of the NK-1-binding ligands, has been exploited to target albumin- and dendrimer-based nanoparticles to GBM and prolong the survival for mice bearing GBM [55,56]. While these studies targeting drug-loaded particles to tumor cells did not explicitly mention the route of entry into the tumors as via the disrupted tumor BBB, it is unlikely that these particles were able to appreciably cross the BBB in tumor regions with an intact BBB. Thus, when targeting the pathologically disrupted BBB, it can be beneficial to enhance drug retention by targeting components of the exposed tumor microenvironment.

## 3. Biochemical Modulation

Pathological BBB disruption is heterogeneous throughout a brain tumor, with brain tumor cells lying behind a relatively intact tumor BBB in the tumor rim in addition to infiltrative cells that can be found outside the tumor margins. Thus, reliance on either passive or targeted delivery to only the pathologically disrupted tumor BBB regions in the glioma core will not eliminate all tumor cells, and a subsequent glioma recurrence will be likely. It therefore remains important to establish strategies that can target the tumor BBB throughout the whole tumor volume. In this section, several methods involving selective biochemical modulation of the tumor BBB are summarized (Table 1).

### 3.1. ATP-Sensitive Potassium Channel Activators

**Figure 2:**
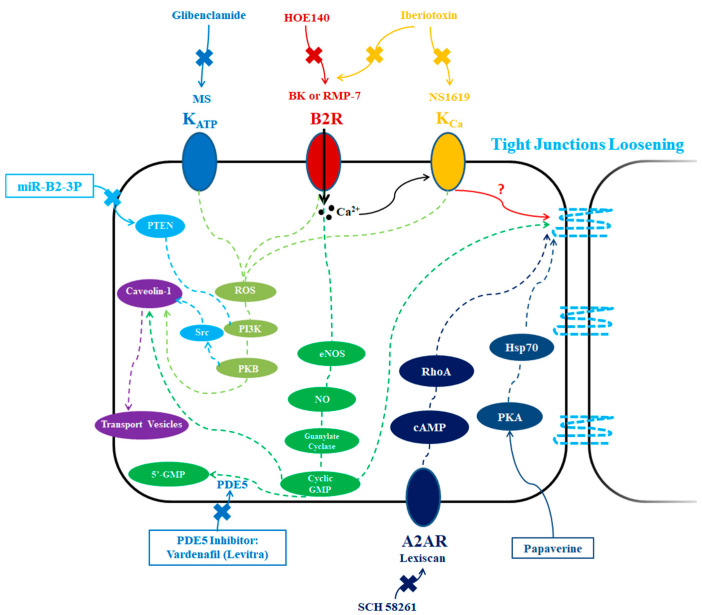
Biochemical modulators of tumor BBB permeability. To selectively open the BBB in the tumor volume, the following biochemical modulators have been applied: ATP-sensitive potassium channel (K_ATP_ channel) activator minoxidil sulfate (MS), calcium-activated potassium channel (K_Ca_ channel) activator NS1619, phosphodiesterase 5 (PDE5) inhibitor vardenafil (Levitra), bradykinin type 2 receptor (B2R) activator bradykinin (BK) or BK analogs such as RMP-7, adenosine 2A receptor (A2AR) agonist Lexiscan, papaverine, and microRNAs such as miR-B2-3P. These effects can be blocked in the presence of several inhibitors, such as glibenclamide for K_ATP_ channel, iberiotoxin for K_Ca_ channel, HOE or iberiotoxin for B2R, SCH58261 for A2AR. The depicted signaling cascades have been suggested to be involved in the regulation of permeability.

Minoxidil sulfate (MS), a selective activator of ATP-sensitive potassium (K_ATP_) channels, was able to selectively increase the tumor BBB permeability in glioma via a transcellular pathway, and could be attenuated by glibenclamide, a selective inhibitor of K_ATP_ channels [57,58] (Figure 2). K_ATP_ channels are overexpressed in tumor vascular endothelial cells with low expression in normal brain endothelial cells, leading to a selective permeability increase in the brain tumor area [57]. In terms of the mechanism for increased tumor BBB permeability, pinocytosis might be involved as increased formation of pinocytotic vesicles was confirmed in both brain tumor endothelium and tumor cells in vivo [57]. Moreover, it has been shown that MS leads to increased caveolin-1 expression at tumor sites, again suggesting that elevated transcellular endocytosis processes could be leading to selective tumor BBB permeability within the tumors [58]. Interestingly, reactive oxygen species (ROS) appeared to be involved in this process, since a ROS scavenger (*N*-2-mercaptopropionyl glycine, MPG) could significantly decrease the effects of MS on caveolin-1 protein expression in the glioma region [58] (Figure 2). Another study using a rat brain glioma (C6) model also identified that MS induced tumor BBB selective opening in the tumor volume in a time-dependent manner by downregulating the expression of TJ proteins, including occludin and claudin-5, and through activation of signaling cascades involving ROS/RhoA/PI3K/PKB [59]. Because of the increased, selective tumor BBB permeability, it has been possible to improve glioma drug uptake for drugs of varying sizes, including anti-HER2 monoclonal antibody and carboplatin, with carboplatin significantly increasing survival in rats bearing RG-2 glioma [57].

### 3.2. Calcium-Activated Potassium Channel Activators

Similarly, intravenous injection of NS1619, an agonist of calcium-activated potassium (K_Ca_) channels, can selectively enhance tumor BBB permeability in glioma, but not BBB permeability in the normal brain tissue [60,61] (Figure 2). Tumor BBB selectivity was thought to be achieved because there is a lower or absent expression of K_ca_ channels in the normal brain tissue area compared with that in tumor and tumor vessels [60,61,62,63]. For example, mRNA and protein analyses showed that K_ca_ channel subunit alpha-1 (KCNMA1) was amplified in 90% of high-grade gliomas samples from patients, as well as in the human high-grade glioma cell line U-87 [63]. However, no amplification of KCNMA1 was found in normal human brain tissues [63]. This suggests that K_ca_ channels could serve as an effective target for selective biochemical tumor BBB modulation and enhance the delivery of chemotherapeutics to glioma [60,61]. For example, the penetration of [^14^C]-carboplatin and anti-HER2 monoclonal antibody into the tumor volume was significantly increased by co-administration of K_Ca_ channel agonist, NS1619, to glioma-bearing Wistar rats via intracarotid infusion, leading to an enhanced survival [61]. In addition, when co-administered with NS1619 via intravenous injection, temozolomide (TMZ) and trastuzumab (anti-HER2 antibody) led to increased survival of mice bearing glioma [64]. The underlying mechanism responsible for NS1619-mediated tumor BBB permeability increases in glioma appears to depend on increased endocytotic, transcellular processes [60,61]. NS1619 treatment increased the number of transport vesicles in the cytoplasm of tumor and brain tumor BBB endothelial cells in vivo, suggesting a role for these transport vesicles in K_Ca_ channel-mediated selective modulation in tumor BBB permeability [60,61,64]. It was found that NS1619 could selectively modulate the protein expression of caveolin-1 through ROS/PI3K/PKB/FoxO1 signaling in brain capillary endothelial cells in tumor area of rat brain glioma (C6) model in a time dependent fashion, resulting in increased caveolae-mediated cholera toxin subunit B endocytosis into tumor microvessels [65] (Figure 2). Interestingly, BBB permeability induced by bradykinin (see below), nitric oxide (NO) donors or an agonist of soluble guanylate cyclase (sGC) could all be attenuated by co-infusion of K_Ca_ channel antagonist, iberiotoxin, indicating K_Ca_ channels might serve as a hub for several strategies used for biochemical modulation of tumor BBB permeability in glioma [60] (Figure 2).

### 3.3. Phosphodiesterase 5 (PDE5) Inhibitors

Cyclic guanosine monophosphate (cGMP), has been shown to be involved in the modulation of vascular permeability [66]. Phosphodiesterase 5 (PDE5) inhibitor can selectively inhibit the degradation of cGMP [67], and the resulting accumulation of intracellular cGMP can result in increased permeability of brain capillaries, particularly in the brain tumor volume [66,68] (Figure 2). PDE5 was found to be strongly expressed in GBM cells isolated from patients [69]. In this way, administration of PDE5 inhibitor was able to selectively modulate the tumor BBB [68]. The tumor uptake of the chemotherapeutic agent, adriamycin, was selectively increased after oral administration of a PDE5 inhibitor, vardenafil (Levitra), without a significant increase in normal brain, leading to a significantly increased survival of rats bearing 9 L gliosarcoma tumors [68]. Similarly, increased cGMP levels were correlated with the enhanced tumor BBB permeability [68]. The selective increase in tumor BBB permeability appears to be induced by enhanced vesicular transport across blood vessels at the tumor margins, and could be attenuated by iberiotoxin, a selective inhibitor for K_Ca_ channels, which are also effectors in cGMP signaling [68]. Moreover, inhibition of caveolae-mediated transcytosis or macropinocytosis could attenuate PDE5 inhibitor-mediated uptake of Herceptin-AlexaFluor-680 in cultured mouse brain endothelial cells, suggesting the possible involvement of these transcellular pathways in PDE5-mediated effects [70].

### 3.4. Bradykinin Type 2 Receptor Activators

Low doses of bradykinin (BK), an activator of bradykinin type 2 (B2) receptors, was demonstrated to selectively and transiently enhance the permeability of the tumor BBB for six tracers with varying molecular sizes in experimental rats bearing RG2 glioma [71] (Figure 2). Importantly, bradykinin does not induce breakdown of the normal BBB unless at very high doses [72]. For instance, 70 kDa dextran had a 12-fold higher permeability in the microvasculature within the glioma in the BK-administered group compared with saline-administered group, while no effect of BK was found for permeability of normal brain microvasculature [71]. RMP-7 (also called cereport or labradimil), the first selective peptide agonist of B2 receptor applied in clinical trials, was confirmed to be able to selectively increase the tumor BBB transport of a various of drugs into brain GBM area in preclinical studies and in clinical trials [73]. The proteolytic lability of BK limited its further application in clinic [74]. Thus, a new analog of bradykinin named retro-inverso bradykinin (RI-BK) was developed, having a 40× higher binding affinity to B2 receptor compared with BK and a resistance to proteolysis [74]. RI-BK could increase the accumulation of coumarin-6-loaded micelles in glioma, but not in normal brain tissue, thus enhancing the anti-glioma effects in mice [74]. The transient BBB opening in the tumor volume mediated by RI-BK was size dependent, with gold nanoparticles of 70 nm having the maximal tumor uptake [75]. As mentioned, the BK- or bradykinin analog-induced tumor BBB opening is transient, usually lasting less than 20–25 min [71,73,76]. The selectivity of BK and BK analog treatment for the tumor region appears to be based on the expression levels of B2 receptors within brain tumor cells [77,78,91] and microvascular endothelial cells in the tumor [79,80]. In clinical isolates, there was high expression of B2R in glioma which increased along with tumor grade [79]. Moreover, a strong immunochemistry staining of B2R was found at both luminal and abluminal surfaces of endothelial cells in tumor microvessels, while the staining of B2R was largely reduced or even absent in microvessels adjacent or outside of the tumor regions [79].

The underlying mechanism for bradykinin-induced permeability increases at tumor sites is a complex process, possibly including transcytosis, TJ loosening, K_ATP_ channels, Ca^2+^ flux, nitric oxide synthase (NOS), NO, and cGMP (Figure 2). The caveolae-1- and caveolae-2-mediated transcellular pathways might be involved in the bradykinin-induced tumor BBB permeability increase in brain tumors [92]. A significantly enhanced expression of caveolin-1 was found after 15, 30 and 60 min RI-BK intra-arterial infusion, suggesting an increase in transcellular transport [75]. In addition, after BK treatment, the expression levels of ZO-1, claudin-5 and occludin were downregulated and the cytoskeleton was rearranged, suggesting an increase in junctional leakiness [81]. RI-BK or BK-induced selective tumor BBB opening in the tumor volume might also be related with the decreased ZO-1 expression and distribution as well as the depolymerization of F-actin [75]. Involvement of accelerated K_ATP_ formation in BK-induced tumor BBB permeability has been reported [93]. BK could also increase intracellular Ca^2+^ level by releasing Ca^2+^ from internal sites or enhancing Ca^2+^ influx [94]. It is possible that the increased intracellular Ca^2+^ might further activate nitric oxide synthase (NOS) and increase NO production. For example, the BK analog, R523, could increase the penetration of various hydrophilic macromolecular agents across the tumor BBB, and the increased permeability could be inhibited by an inhibitor of nitric oxide (NO) synthase, L-NA [80,95], suggesting a complex underlying mechanism. In addition, the effects of BK could be attenuated by pretreating with a NOS inhibitor, L-NAME [96]. Decreased cGMP formation induced by an inhibitor of soluble guanylate cyclase (sGC), LY83583, could block BK- induced BBB permeability [97]. Conversely, NO could also activate sGC, further increasing the formation of cGMP [98], indicating NO might be a core factor in the modulation of BK-induced BBB permeability [80,99]. Furthermore, NO-producing agents, including L-arginine and hydroxyurea, could increase tumor permeability in rats bearing 9 L gliosarcoma, and involved the change of eNOS and cGMP levels in the tumor region [100]. The findings suggest that use of oral NO donors may be a strategy to enhance the delivery of chemotherapeutics to malignant brain tumors [100].

### 3.5. Adenosine 2A Receptor Activators

Adenosine is an endogenous ligand for the adenosine 2A receptor (A2AR) which belongs to the family of G protein-coupled receptors [82]. Activating the vascular A2AR signaling pathway using Lexiscan, an FDA-approved selective A2AR agonist, can significantly and transiently enhance BBB permeability in mice and this permeability is attenuated in transgenic mice lacking A2AR [82,83] (Figure 2). Importantly, it was found that A2AR expression was higher in human glioma margins than that of peritumor normal brain area, suggesting it may be possible for A2AR agonists to selectively alter tumor BBB permeability in glioma patients [20]. In addition, in preclinical studies, A2AR agonists produced no obvious side effects even after multiple repeats of BBB modulation [84,85]. However, although Lexiscan has been shown to be effective in rodents to improve the BBB penetration of agents with various sizes via activating A2AR, limited success has been achieved in clinic [101,102]. For example, Lexiscan (0.4 mg/kg) failed to increase temozolomide concentrations in the brain or GBM volume in human patients [101], and more efforts to explore alternative doses and schedules might be warranted. In terms of mechanism, TJ and cytoskeletal proteins are of vital importance in regulating A2AR-mediated BBB permeability [82]. For example, A2AR activation and commensurate increased BBB permeability were mainly mediated by the downregulation of junctional proteins such as VE-cadherin and claudin-5, as well as the reorganization of cytoskeletal actin, which was related with cAMP/RhoA signaling [82,83].

### 3.6. Papaverine

Papaverine is a natural opioid that can cause a reversible increase in tumor BBB permeability after intra-arterial infusion [86,87,88]. In particular, Evans blue and ^14^C sucrose extravasation were increased after papaverine infusion, and recovered to control levels after 5 h, indicating that papaverine could temporally open the BBB, particularly in the brain tumor region when using a C6 glioma rat model [86,89]. An enhanced tumor uptake of chemotherapeutic, Bis-chloronitrosourea, was achieved for patients with malignant glioma when combined with papaverine [88]. The involvement of protein kinase A (PKA) and heat shock protein 70 (HSP70) has been demonstrated to be involved in this reversible increase in tumor BBB permeability in rats bearing C6 glioma [89] (Figure 2). A combination of western blotting and immunochemistry analysis also demonstrated that papaverine could downregulate claudin-5, occludin and F-actin expression by tumor microvessels, but not in normal brain tissue, suggesting that the tumor BBB opening was due to TJ dysfunction [89].

### 3.7. microRNAs

Recently, microRNAs (miRNAs) have been identified as important regulatory factors for various biological processes, including vascular permeability. In particular, miR-132-3p expression was found to be upregulated in glioma endothelial cells, and therefore could be a novel target for selective tumor BBB modulation in brain tumors [90]. Researchers have further identified miR-132-3p as an important miRNA in selectively increasing tumor BBB permeability in glioma and overexpression of this miRNA yielded increased uptake of doxorubicin in rat C6 glioma tumors [90]. In vitro studies indicated that endocytosis of cholera toxin subunit B and FITC-bovine serum albumin were significantly increased by miR-132-3p, via PTEN/PI3K/PKB/Src/Caveolin-1 signaling pathways [90] (Figure 2).

While the aforementioned biochemical strategies to control tumor BBB permeability have in many cases exhibited selective increases in BBB permeability and commensurate increases in drug uptake, clinical success has been limited. More potent drugs or targeting ligands are likely needed to pair with these approaches. In addition, it may also be possible that the combination of these biochemical approaches for tumor BBB modulation with selective targeting of the exposed tissue as described in the Pathological BBB Disruption section could enhance efficacy.

## 4. Physical Modulation

Another strategy to target BBB permeability to tumor regions is the use of physical disruption methods that employ various forms of electromagnetic radiation and ultrasound. These approaches can be widely applicable for drug delivery and have been proposed to efficiently and selectively allow drug passage into the tumor margin with minimal damage, including in several clinical applications (Table 2).

### 4.1. Electromagnetic Pulse (EMP)

Radiofrequency electromagnetic radiation (EMP) has been shown to increase the permeability of BBB for agents that normally do not pass the BBB such as Evans blue [103,104]. The induced permeability appears reversible as EMP exposure of an in vitro BBB model consisting of cocultured primary rat brain microvascular endothelial cells and primary rat astrocytes increased permeability and the effect was normalized after 24 h [105]. Further, in vivo studies have demonstrated that EMP exposure was able to transiently disrupt the BBB in healthy rats, with BBB permeability increased at 1 h, peaking at 3 h, and recovering at 12 h using Evans blue, endogenous albumin and lanthanum nitrate as tracers [106,107]. The chemotherapeutic 1-(2-chlorethyl)-cyclohexyl-nitrosourea (CCNU, lomustine) could be delivered to the brain tumor volume when glioma-bearing rats were exposed to EMP, enhancing tumor toxicity without significant side effects in normal brain tissue [108]. Although whole body exposure is currently used to administer EMP, there is some evidence of tumor BBB selectivity [108], and further work is required to determine the basis of such selectivity. In terms of mechanism, in vitro BBB permeability modulation by EMP was shown to be mediated by alteration in protein expression levels of the ZO-1 and claudin-5 TJ proteins [105]. EMP exposure in healthy rats also significantly decreased the gene and protein expression of TJ proteins, occludin and ZO-1, and increased the expression of matrix metalloproteinase such as MMP-2 and MMP-9, which were suggested to drive the observed changes in BBB permeability [107].

### 4.2. Laser-Induced Thermal Therapy (LITT)

Laser-induced thermal therapy (LITT) is another strategy being applied in treating brain tumors and employs a stereotactically implanted laser source within the tumor volume. Currently, LITT has been used in a thermal ablation modality, and pilot clinical studies have confirmed its effectiveness and feasibility in patients suffering recurrent brain tumors, with therapeutic effects monitored by real-time MRI [109]. In addition, it has been shown that LITT can induce a local, selective opening of the healthy BBB as demonstrated by extravasation of Evans blue, fibrinogen and IgM, and LITT further facilitated the passage of anti-tumor drugs such as paclitaxel into rat brains [110]. The BBB was observed to be opening at 1.5 h after LITT and it reached the peak value at 2.5 h after LITT [110]. It has been suggested that membrane defects in the capillary endothelium during LITT might be the direct cause of BBB opening and that these defects might be related to LITT-induced hyperthermia [110]. Other studies have also demonstrated the possible influence of hyperthermia on BBB integrity, showing that the breakdown can occur as brain temperatures rise over 41 °C [111,112]. It has also been suggested that polyamine synthesis could play a role in LITT-induced BBB opening as hyperthermia has been shown to stimulate increased polyamine synthesis [110,113]. Heat stress can also cause BBB opening that is related to a significant upregulation of NOS activity in the CNS [114]. To date, there have been no reports of specific changes in TJ proteins upon LITT-induced BBB opening [137]. While LITT has been demonstrated to mediate a local, selective healthy BBB opening, it has yet to be tested for selective tumor BBB opening and potential improvements in tumor drug uptake.

### 4.3. Radiotherapy: Synchrotron Microbeam Radiation Therapy (MRT)

Conventional radiotherapy can increase BBB permeability in preclinical animal models and clinical trials [115,116,117], enhancing the efficacy of systemic administration of chemotherapeutic drugs in treating brain tumors such as glioma [116,118,119]. In a clinical study, irradiation with 20 to 40 Grays induced tumor BBB opening and facilitated the entry of chemotherapeutics such as methotrexate in glioblastoma patients, increasing survival time [117]. Apart from conventional radiotherapy, microbeam radiation therapy (MRT) is a different form of radiotherapy that has been used to alter the tumor BBB that relies on radiation delivery strategies to focus the irradiation to the tumor site [120,121]. In this way, MRT has been shown to selectively attenuate or even block tumor growth in several tumor models, leading to an increased animal survival with a high tolerance of normal tissues to MRT [120,122]. Interestingly, from the point of view of this review, exposure to MRT was shown to make the tumor BBB more permeable to low-molecular-weight contrast medium [119], and MRT therapy did not modulate the BBB permeability in contralateral brain, but selectively increased the tumor BBB permeability in a rat model of intracranial GBM [123]. Real-time MRI also demonstrated that Gd-DTPA uptake could be increased in the initially non-enhanced tumor area but not in the surrounding normal brain tissue during MRT, indicating selective effects on the tumor BBB [138]. Although the underlying mechanism responsible for increased MRT-induced tumor BBB permeability is not yet clear, conventional radiation has been associated with the decreased expression and rearrangement of TJ proteins including claudin-5, ZO-1 and beta-catenin [115]. Despite the aforementioned evidence that MRT can lead to a selective increase in tumor BBB permeability, the strategy has yet to be combined with co-administration of a chemotherapeutic and efficacy demonstrated in a brain tumor model.

### 4.4. Focused Ultrasound (FUS)

**Figure 3:**
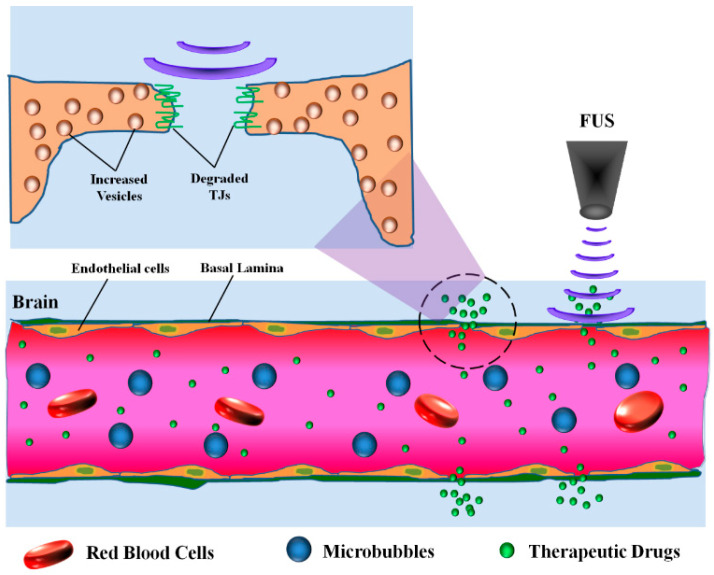
Schematic of BBB disruption by focused ultrasound (FUS) and drug uptake into disrupted regions. Inset indicates that when microbubbles apply mechanical forces on endothelial cells, tight junctions can open and there can be increased transport vesicle formation.

FUS has been rapidly developing as a promising noninvasive method for localized BBB opening for treatment of CNS diseases (Figure 3) [139,140]. The physical interactions between systemically administered microbubbles and focused ultrasonic waves enable the transient and reversible disruption of the BBB in targeted brain regions [125,126]. To date, FUS has been applied for enhancing tumor BBB penetration of anti-tumor drugs such as doxorubicin, temozolomide, carboplatin and paclitaxel into glioma [20,127,128,129]. For example, regardless of its anti-glioma potency, paclitaxel has showed little benefit in vivo due to its poor BBB penetration, and FUS was shown to increase paclitaxel penetration across the tumor BBB and improve its anti-glioma effects [130]. A clinical FUS device was developed and tested for the delivery of carboplatin to the brain in rats bearing F98 glioma model, leading to 1.7-fold and 3.3-fold higher MRI contrast agent signal in the center and margin of tumor, respectively, compared to tumor without FUS [131]. FUS can also increase tumor uptake of biologicals and complex nanostructures such as IgG and gold particles [141,142,143,144,145], and has been shown to be a relatively safe, well-tolerated approach for healthy BBB and tumor BBB opening in preclinical animal models [132,133], and in clinical trials where improved drug efficacy was observed [129,134]. Given that FUS is showing early promise in terms of clinical implementation, safety is being more extensively evaluated. FUS-induced barrier opening can last for several hours, with a barrier restoration time that depends on the molecular size of the tracer or therapeutics [125]. Although the extravasation of albumin and red blood cells can be seen in FUS treated regions, they can be rapidly cleared by glia and it has been suggested that FUS does not lead to ischemia or adverse behavioral effects [146]. However, microbubble oscillation induces mechanical shear forces that might still be of potential risk, and cause excessive immune reactions and brain hemorrhage [132,147]. It is also been reported that unpredictable hot spots might exist in brain regions receiving FUS [148]. In terms of mechanism, FUS can lead to upregulation of cellular machinery in charge of transcellular transport, including caveolin-1 showing a peak value at 1 h after sonication, with a commensurate downregulation of TJ proteins such as claudin-1, claudin-5 and occludin [135]. Indeed, both transcytosis and free passage via the affected BBB endothelial cells have been shown to be involved in FUS-mediated BBB opening, and the mechanisms may be dependent on the intensity of the ultrasound [126].

## 5. Conclusions and Future Perspectives

Existing as an interface between the blood and brain tumor tissue, the tumor BBB hinders the glioma uptake of many therapeutics. While methods to globally open the BBB have been proposed to enhance drug penetration into the brain tumor volume, they are often met with challenges resulting from their non-specific nature of BBB opening, which could damage normal brain tissue. By contrast, the methods described here have the potential to selectively and efficiently modulate the tumor BBB in glioma. Although pathological disruption does partially expose regions of the brain tumor core to the systemic circulation, the extent of disruption can be heterogeneous across the tissue, and tumor cells may reside behind a relatively intact tumor BBB. While we believe that targeting and retention of therapeutic cargo in the disrupted tumor BBB regions can clearly lead to improved glioma treatment, it will likely fail to eradicate all tumor cells, particularly in the tumor rim. Thus, such approaches will need to be combined with biochemical or physical approaches to further selectively open the tumor BBB. Importantly, blood vessels in tumor tissue can have differential molecular expression profiles compared with normal brain blood vessels and biochemical modulators could potentially take advantage of these differences for selective BBB opening. The search for additional pathways that can be used to selectively modulate tumor BBB permeability especially at the tumor margins should continue, as those described here have shown limited clinical success despite being investigated for over 20 years. In addition, several physical methods can be used to stereotactically target the brain tumor volume, thereby generating localized BBB disruption both within and surrounding the tumor volume. It is our opinion that FUS, in particular, offers a wealth of opportunities for enhanced glioma delivery, especially given the strides that have been made to evaluate its clinical safety. Regardless of choice, if the tumor BBB can be reversibly disrupted throughout the entire tumor volume by biochemical or physical means, the various structural and cellular components normally invisible to the circulation, including ECM, can be exposed. Targeting of exposed components may allow therapeutic accumulation throughout the entire tumor volume. Moving forward, one could envision combining new and improved targeted therapy strategies with optimized strategies of BBB disruption to more selectively and efficaciously deliver medicine to those suffering from glioma.

## Figures and Tables

**Table 1:** Selective biochemical modulation for circumventing the tumor BBB in treating glioma.

Biochemical Modulation	Tumor Enriched Expression	Applied Drugs	FDA Approved	Tight Junction Effects	Vesicular Transport Effects	Clinical Stage	Refs
ATP-sensitive potassium channel (K_ATP_ channel)	Yes	Minoxidil sulfate	Yes	Occludin↓, Claudin-5↓	Transport vesicles↑, Caveolin-1↑	Preclinical	[57,58,59]
Calcium-activated potassium channel (K_Ca_ channel)	Yes	NS1619	No	_	Transport vesicles↑, Caveolin-1↑	Preclinical	[60,61,62,63,64,65]
Phosphodiesterase 5 (PDE5)	Yes	Vardenafil (Levitra)	Yes	_	Transport vesicles↑	Preclinical	[66,67,68,69,70]
Bradykinin type 2 receptor (B2R)	Yes	Bradykinin and analogs	No	ZO-1↓, Occludin↓, Claudin-5↓	Caveolin-1↑	Clinical	[71,72,73,74,75,76,77,78,79,80,81]
Adenosine 2A receptor (A2AR)	Yes	Lexiscan	Yes	Occludin↓, Claudin-5↓	_	Clinical	[20,82,83,84,85]
Papaverine	_	_	No	Occludin↓, Claudin-5↓	_	Clinical	[86,87,88,89]
microRNAs	_	miR-132-3p	No	_	Caveolin-1↑	Preclinical	[90]

Note: Up-regulated ↑; Down-regulated ↓.

**Table 2:** Selective physical modulation for circumventing the BBB in treating glioma.

Physical Modulation	Physical Source	Invasive or Noninvasive	Tight Junction Effects	Vesicular Transport Effects	Clinical Stage	Refs
Electromagnetic pulse (EMP)	Electromagnetic radiation	Noninvasive	ZO-1↓, Occludin↓, Claudin-5↓, MMP-2↑, MMP-9↑	_	Preclinical	[103,104,105,106,107,108]
Laser-induced thermal therapy (LITT)	Laser	Invasive, under anesthesia	_	_	Clinical	[109,110,111,112,113,114]
Radiotherapy: Synchrotron microbeam radiation therapy (MRT)	X-ray beams	Noninvasive	ZO-1↓, Claudin-5↓, beta-catenin↓	_	Preclinical	[115,116,117,118,119,120,121,122,123,124]
Focused ultrasound (FUS)	Ultrasonic waves with microbubbles	Noninvasive	Occludin↓, Claudin-1↓, Claudin-5↓	Transport vesicles↑, Caveolin-1↑	Clinical	[125,126,127,128,129,130,131,132,133,134,135,136]

Note: Up-regulated ↑; Down-regulated ↓.
